# Preparing for a pandemic: spending dynamics and panic buying during the COVID‐19 first wave

**DOI:** 10.1111/1475-5890.12271

**Published:** 2021-06-08

**Authors:** Martin O'Connell, Áureo de Paula, Kate Smith

**Affiliations:** ^1^ Institute for Fiscal Studies; University College London

**Keywords:** panic buying, hoarding, coronavirus

## Abstract

In times of heightened uncertainty, consumers face incentives to build up precautionary stocks of essential supplies. We study consumer spending dynamics during one such time, the first infection wave of the COVID‐19 pandemic, using household scanner data covering fast‐moving consumer goods in the United Kingdom. We document large increases in demand for storable products, such as food staples and household supplies, in the days before lockdown. Households in all socio‐economic groups exhibit unusually high demand pre‐lockdown, but there is a clear gradient, with the largest demand spikes for wealthier households. Although stories of people purchasing extreme amounts received a lot of attention, higher aggregate demand was mainly driven by more households than usual choosing to buy storable products, with only small increases in average quantities bought on a given trip. Temporary limits on the number of units per transaction, introduced following the demand spike, are therefore unlikely to lead to the avoidance of stock‐outs.

## 1 INTRODUCTION

The prospect of supply disruptions, restrictions on movement, or fear of demand‐driven shortages provides a rationale for people to build up precautionary stocks of essential supplies. This was evident during the early phase of the COVID‐19 pandemic, with widespread reports of panic buying and subsequent shortages of popular goods.^1^ This led to calls from politicians for people to behave responsibly,^2^ supermarkets imposing limits on the quantity of individual products that consumers were permitted to buy per visit, and the UK competition authority relaxing competition rules.^3^ Several papers that use financial transaction data to track the effect of the pandemic on consumer spending highlight a pre‐restriction spike in grocery spending,^4^ and Keane and Neal (2020) document sharp increases in Google searches for terms such as ‘panic buying’ and for necessities such as ‘toilet paper’ across many countries. However, we know relatively little about the types of products that were hoarded, nor the changes in purchasing behaviour that led to demand spikes.

In this paper, we are, to the best of our knowledge, the first to use household‐level scanner data to document purchase dynamics during the first wave of the COVID‐19 pandemic. Using data for the United Kingdom, we show that there were substantial spikes in demand for storable products, and this was primarily driven by many more households than usual choosing to buy these products in the run‐up to lockdown, with only small increases in the average quantities bought per transaction. Our results provide valuable evidence that is relevant for the immediate challenge of dealing with further bouts of hoarding that may precede more stay‐at‐home orders, and contribute to our broader understanding of the way that people build up precautionary stocks in times of significant uncertainty. In particular, we show that neither quantity limits nor requests to ‘behave responsibly’ are likely to have a large impact on mitigating the size of demand spikes. This is because dramatic spikes in demand can be – and were, in the setting that we study – driven by many households buying more frequently than usual, rather than excessive purchases by a small number of households.

We document purchase dynamics using household‐level scanner data, covering fast‐moving consumer goods. We use data on the purchases of a nationally representative panel of 17,000 households. Each participant records all purchases they make and bring into the home, including from brick‐and‐mortar stores and online, at the UPC (barcode) level. These data have a number of key advantages for measuring hoarding behaviour over other real‐time data sets covering consumer spending. First, we observe the disaggregate products that consumers purchase. This allows us to document precisely which product categories drove the surge in demand. Second, the data contain information on quantities and number of packs bought (as well as expenditure). Therefore, we are able to track purchase incidence, pack size and number of units at the daily frequency. This enables us to assess the bite of limits placed on the number of units that consumers are allowed to buy at any one time. Third, the long purchase history for households allows us to compare their behaviour during the pandemic with normal times. The data cover the subset of consumer goods (roughly groceries and household supplies) among which reports of panic buying were concentrated. Using this data set, we establish four sets of results regarding consumer purchase dynamics in the run‐up to the UK's first national lockdown on 23 March 2020.

First, we show that there were large spikes in spending on storable products in the four weeks preceding lockdown. Spending on staples (such as canned goods, pasta, rice and grains) rose sharply at the end of February, peaking on 14 March at over 80 per cent above the level on the same day in 2019. A similar pattern is evident for non‐food household supplies (such as soap, cold treatments and toilet tissue), with demand peaking at nearly 75 per cent above the previous year's levels on 14 March. Spending on the remaining set of fast‐moving consumer goods (discretionary calories and perishable foods) increased much more gradually into March, and continued to rise as the UK entered the lockdown period. We show that the spike in spending on staples and household supplies in the run‐up to lockdown was driven by most households increasing their demands rather than by a small number of extreme purchasers.

Second, we identify which product categories were the primary drivers of these increases in spending and the extent to which quantity spikes at the category level were driven by changes in purchase frequency (extensive margin) or in the average quantity bought conditional on purchasing (intensive margin). Thirty, out of a total of 138, product categories experienced an increase in demand of more than 25 per cent; these categories account for 70 per cent of the increased spending on household supplies and 53 per cent of the increase for staples. Across these categories, on average, 70 per cent of the demand increase was due to increased purchase frequency and the remainder was due to households buying larger amounts conditional on purchasing. The increase in purchase frequency was relatively more important for those categories that experienced the largest increases in demand. We show that the increase at the extensive margin was mainly driven by more households than normal choosing to buy these categories once over the ‘hoarding period’ preceding the lockdown in late March, rather than by an increase in the number of times purchasing households bought them. We also show that, although there was a small increase in the number of trips that households made to the shops over this period, this increase was not enough to explain the higher purchase frequency for storable categories. Instead, households were more likely to buy storable products when they did visit a store. This suggests that even in times when households are shopping less frequently than normal, spates of hoarding remain a possibility.^5^

Third, we show that across socio‐economic groups, there was a sharp increase in the quantity purchased of all storable categories, underlining that hoarding was a widespread phenomenon. However, the average increase was substantially bigger for households with higher socio‐economic status (SES): those in the top group increased purchases by 54 per cent across the affected categories, compared with 33 per cent for the bottom group. We show that this pattern is entirely driven by differences along the extensive margin, with higher‐SES households increasing their probability of buying by more than lower‐SES households. Wealthier households were therefore able to build up larger precautionary stocks than less well‐off households. Cox et al. (2020) find that, although high‐income households in the US experienced a greater decline in spending *following* the introduction of restrictions, this was entirely driven by differences in *non‐essential* spending. Our analysis sheds light on the changes in purchasing behaviour that drove the spike in spending on essential groceries *prior* to the introduction of restrictions that Cox et al. (and others) have documented.

Fourth, we consider the likely impact of limiting the number of units of each product that households are permitted to purchase on a visit to the store. These limits were imposed by supermarkets following the demand spike just prior to the UK's lockdown. We show that it is unlikely that they would have prevented the large spikes in demand seen in many categories. Had a limit of two units been in place for the four weeks up to lockdown, and had households made no adjustment to their behaviour to circumvent the constraint, the average increase in quantity in the top 30 categories would have been 34 per cent rather than 44 per cent. Among the eight categories that experienced spikes of more than 50 per cent, the average quantity increase would have been 55 per cent rather than 66 per cent. This suggests that reintroducing limits may have only a modest impact on preventing a further round of hoarding and shortages.

We build on and contribute to a fast‐growing literature that uses a range of real‐time data sets, including financial transaction, survey and publicly available data, to document the impact of the pandemic on economic activity.^6^ A contribution of this literature is to show how aggregate expenditure, as well as spending in broad sectors of the economy, has evolved over the pandemic, and how this varies across income groups. For example, Baker et al. (2020a) and Cox et al. (2020) describe how consumer spending in the US has changed over the pandemic; they highlight a pre‐restriction spike in grocery spending. Chronopoulos, Lukas and Wilson (2020), Davenport et al. (2020) and Hacioglu, Känzig and Surico (2020 and 2021) document a similar pattern for the UK. However, the nature of the data used in these studies means that they are not able to identify which goods drove the spike in spending, nor whether it was driven by changes at the extensive or intensive margins of purchasing. We complement this work by studying purchase dynamics in a sector of the economy that was particularly hard hit by bouts of hoarding in the first phase of the pandemic. We exploit granular, household‐level scanner data to describe the changes in behaviour that drove the large spike in grocery spending prior to lockdown. This allows us to provide valuable evidence on the nature of hoarding and, notably, that it was primarily driven by more people than usual buying a number of key categories rather than by a small number of shoppers buying extreme amounts.

We also contribute to the wider literature that studies panic buying in other settings – for example, in response to weather shocks and natural disasters. Consistent with our results, Hori and Iwamoto (2014) find that the hoarding following the 2011 earthquake in Tohoku, Japan, was primarily due to an increase in the share of people buying. Hansman et al. (2020) study hoarding behaviour during the 2008 Global Rice Crisis, highlighting the role of sticky prices as a motivating factor for households to stock up. Like us, they find that hoarding behaviour was more prevalent among richer households. Croson et al. (2014) study the motivations for hoarding in an experimental study of ‘the beer distribution game’, which removes the incentives to hoard due to price expectations, demand uncertainty and horizontal competitive effects. They find that, despite there being no rational motivations to hoard, there is considerable over‐ordering; their results are robust to excluding a number of outliers that reflect drastic increases in ordering. A key finding of our analysis is that a lot of people buying a little bit more can lead to substantial increases in aggregate demand and subsequent shortages.

The paper is structured as follows. We outline the data set in Section 2 and document spending dynamics across broad sets of products in Section 3. In Section 4, we explore how widespread hoarding was across disaggregate product categories and households. In Section 5, we discuss some lessons for policy. Section 6 concludes and we present additional tables and figures in an online appendix.

## 2 DATA AND SETTING

### 2.1 Data set

We use household‐level scanner data collected by the market research firm Kantar's FMCG Purchase Panel. The data cover purchases of fast‐moving consumer goods (FMCG), which include all food and drinks (including alcohol), as well as toiletries, cleaning products and pet foods, that are brought into the home by a representative sample of households living in Great Britain (i.e. the UK excluding Northern Ireland).^7^ Kantar recruits panellists to match the demographic profile of the Great British population, targeting the recruitment of households on the basis of age, social class, size of household and UK region of residence. Households are rewarded for their participation in the survey with monetary vouchers redeemable at stores not covered by the purchase panel. Participating households record purchases at the UPC (or barcode) level using hand‐held scanners. The data set includes purchases made in both brick‐and‐mortar stores and online. For each transaction, we observe quantity, number of units, expenditure, price paid, store and UPC characteristics.

We use data covering the period 1 January 2019 to 9 August 2020. Our sample is a balanced panel of 17,093 households who record their purchases over this period. In Table A.1 in the online appendix, we show that the sample is comparable along key demographic dimensions to the UK's main consumer spending survey – the Living Costs and Food Survey (LCFS).

Recent research studying consumer spending over the pandemic has used data sources other than the main consumer spending surveys, in part because official survey data covering the pandemic period have not yet been released. In the UK, these other data sources include the Kantar (household scanner) data, which we use here, as well as the financial transaction data collected via the Money Dashboard budgeting app. These data have a number of advantages, including their timeliness and longitudinal nature, but they also have some disadvantages. Previous research comparing the spending patterns in the Kantar and LCFS data sets^8^ shows that spending is somewhat lower in the Kantar data than in the LCFS, in part due to lower recording of non‐barcoded items.^9^ However, these papers also show that spending patterns across demographic groups and product categories match closely, and spending differences are stable over time. In Figure A.1 in the online appendix, we show that spending on food and non‐alcoholic beverages evolves similarly in the LCFS and Kantar data over the period 2011 to 2018.

As well as matching the broad trends in spending over the past decade, it is also important that household recording behaviour does not change in the period we study, during the run‐up to the UK's first national lockdown on 23 March 2020. In Figure A.2 in the online appendix, we show that total spending on groceries recorded in the Kantar data peaked two weeks before the beginning of lockdown and fell back to 2019 levels immediately following the onset of lockdown, before rising to a level above that in 2019. This pattern is similar to that documented by Chronopoulos, Lukas and Wilson (2020) and Davenport et al. (2020) using Money Dashboard data.

A significant strength of our data is that they contain information on households' purchases for a substantial period prior to the pandemic and we are therefore able to compare their behaviour during the pandemic with their behaviour in normal times. In addition, the granularity of the scanner data allows us to analyse changes in purchasing patterns for disaggregate product categories, as well as whether these changes are driven by increases in purchasing frequency or buying more on a given shop. This analysis would not be possible with more aggregated consumer spending surveys.

### 2.2 Timeline

The first case of COVID‐19 was recorded in the UK on 30 January 2020. There was an acceleration in case numbers across the globe during February. In Europe, the first ‘lockdowns’ (or ‘stay‐at‐home’ orders) were introduced in the Lombardy region of Italy on 23 February and were extended to the rest of the country on 9 March. By 3 March, when the UK government first published its strategy for responding to the pandemic – the ‘coronavirus action plan'^10^ – there were a total of 49 confirmed cases in the UK. A rapid increase in case numbers resulted in the government introducing a nationwide lockdown on 23 March. The lockdown entailed closure of all non‐essential businesses; however, businesses specialising in the sale of fast‐moving consumer goods, such as supermarkets, convenience stores and off‐licences, were permitted to remain open. On 11 May, England moved into the ‘Stay Alert’ phase, with the government no longer encouraging people to stay at home. From this point forward, lockdown restrictions were gradually lifted.

## 3 PURCHASE DYNAMICS

**FIGURE 1:**
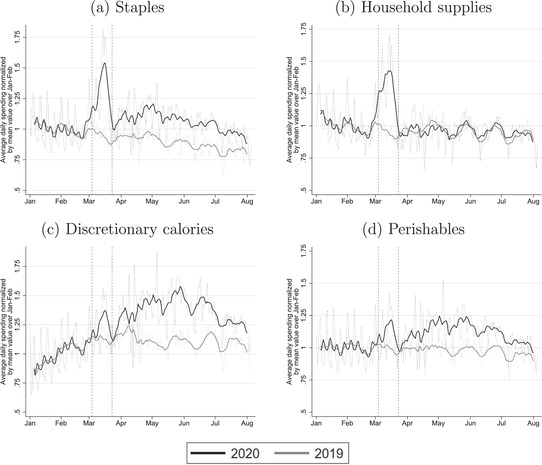
Aggregate spending *Note*: The panels show total daily expenditure on staples, household supplies, discretionary calories and perishables. See Table A.3 in the online appendix for a list of the product categories in each grouping. The dotted lines show daily expenditure after removing day of the week and holiday effects. The solid black line shows a nine‐day moving average through these points. In each case, the line is normalised by the mean value over January and February. The vertical dashed lines indicate the announcement of the UK's coronavirus action plan on 3 March and the beginning of lockdown on 23 March.

In this section, we document purchase dynamics over the pandemic for four broad groups of products that together comprise all fast‐moving consumer goods. For each group, Figure 1 shows the evolution of daily expenditure between 1 January and 9 August in 2019 and 2020. The two vertical dashed lines denote the day in 2020 that the coronavirus action plan was published and the beginning of the first lockdown.

Panel a focuses on ‘staples’. These products are storable and include canned goods, rice and grains.^11^ This product group accounts for 16 per cent of total fast‐moving consumer goods expenditure in 2019. The graph shows that spending evolved similarly in 2019 and 2020 up until the end of February. At this point, spending in 2020 rose sharply, peaking on 14 March at over 80 per cent above the previous year's level. Spending then fell back to close to normal levels at the beginning of lockdown. After lockdown began, spending rose gradually before settling at an elevated level compared with the beginning of the year and with 2019, but considerably below the peak in March.

Panel b focuses on ‘household supplies’, which cover all non‐food‐and‐drink fast‐moving consumer goods – such as soap, cleaning products and toiletries – and comprise 14 per cent of spending in 2019. The evolution of daily expenditure follows a similar pattern to staples up until the beginning of lockdown: expenditure is very similar in 2019 and 2020 until the end of February, at which point spending in 2020 rises sharply, peaking at nearly 75 per cent above the previous year's levels on 14 March. Unlike staples, expenditure on household supplies in 2020 was similar to 2019 from the beginning of lockdown onwards.

Panels c and d show the paths of expenditure on ‘discretionary calories’ – for example, alcohol, desserts, confectionery and soft drinks – and ‘perishables’ – i.e. fresh food such as fruit, vegetables, meat and dairy. These account for 27 per cent and 42 per cent, respectively, of fast‐moving consumer goods spending in 2019. For both of these groups of products, the spike in daily expenditure in March is smaller than the increase in spending during lockdown.

Figure 1 makes clear there was a large spike in spending on staples and household supplies in the run‐up to lockdown. Following the spike, at the onset of lockdown, spending on household supplies returned to a level similar to that in 2019. Spending on staples dipped at the beginning of lockdown, though remained at a level higher than at the same time in 2019, before moderately rising during lockdown. Spending on discretionary calories and on perishables was also higher during lockdown than at the same time in 2019. This higher spending is predominantly due to increased demand during lockdown as households worked from home and switched away from shut‐down restaurants. In addition, a spike in inflation for fast‐moving consumer goods in the first week of lockdown, driven by fewer promotions (documented in Jaravel and O'Connell (2020b)), contributes toward higher spending during the lockdown period.^12^ However, the increases in spending on staples and household supplies during lockdown are dwarfed by the March spikes.

**FIGURE 2:**
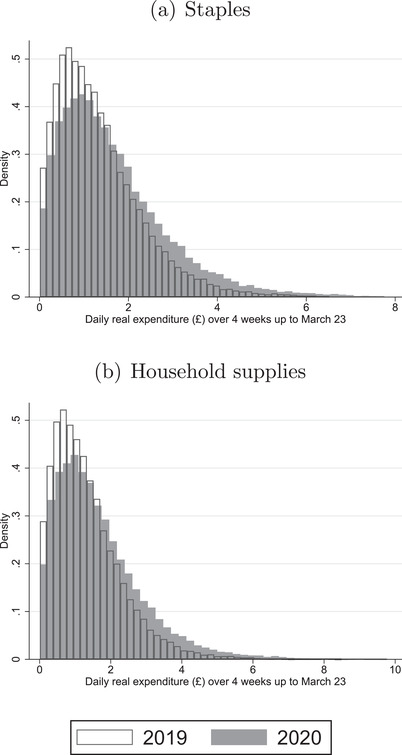
Distribution of real spending in four weeks up until 23 March *Note*: For each household, we compute its average daily real expenditure on staples and household supplies over the four‐week period ending 22 March in both 2019 and 2020. The panels show the distributions in 2019 and 2020 for staples (panel a) and household supplies (panel b). See Table A.3 in the online appendix for a list of the product categories in each grouping. Daily real expenditure is constructed holding UPC prices fixed at their average level over the four‐week period ending 22 March in 2019.

Press reports over the four‐week period prior to lockdown suggest that a small number of greedy consumers were leading to stock‐outs.^13^ In Figure 2, we show how the distribution of household spending on staples and household supplies changed in the run‐up to lockdown. This allows us to assess whether it was the case that a small number of extreme purchasers drove the spike in aggregate spending on staples and household supplies.

Panel a of Figure 2 focuses on staples. It shows the distribution of average daily *real* expenditure on staples in the four‐week period up until 23 March across households in both 2019 and 2020.^14^ For each distribution, we hold UPC prices at their 2019 level, so that the graph is not influenced by price inflation. The graph shows that there was a rightward shift in the distribution in 2020 relative to 2019. Panel b, which focuses on household supplies, shows a similar rightward shift in the distribution of real expenditure. Together they point towards a moderate increase in demand for staples and household supplies by many households, rather than a small number of extreme purchasers, driving the spike in aggregate spending. This does not rule out that extreme purchasing was important at the more disaggregate level of product categories. We turn to the analysis of purchase dynamics at the product category level in the next section.

## 4 HOW WIDESPREAD WAS HOARDING?

In this section, we explore how widespread hoarding was, both across disaggregate product categories and across households. We document the size of quantity spikes across product categories, assess the relative importance of changes in the probability of purchasing and quantity conditional on buying, and provide evidence on the extent to which the size of households’ demand increase in one category correlates with their increase in purchases in other categories. We then document heterogeneity in demand spikes across households from different socio‐economic groups.

### 4.1 Which product categories drove the demand spike?

We use the granular nature of our data to provide evidence on the product categories that experienced the largest spikes in demand, and the extent to which this contributed to the overall spikes in spending shown in the previous section. We study purchase dynamics across 138 product categories; see Table A.3 in the online appendix for a list. For each category, we calculate the percentage change in average daily quantity purchased over the four‐week period running up to the start of lockdown on 23 March, relative to the same period in 2019.

**FIGURE 3:**
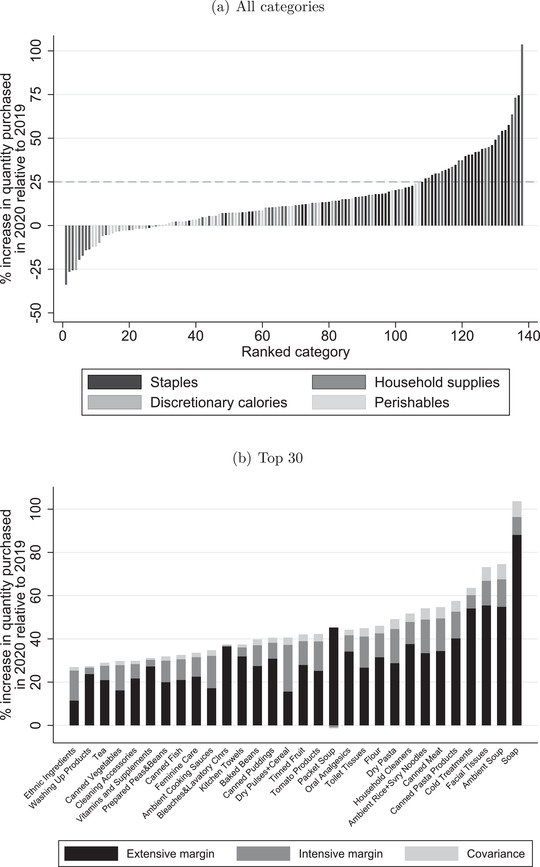
Quantity increases across categories in four weeks up until 23 March *Note*: For each category, we compute the mean quantity purchased (across household‐days) over the four‐week period ending 22 March in 2019 and 2020. The bars show the percentage change between these two periods. Panel a shows changes for all categories. See Table A.3 in the online appendix for a list. Panel b zooms in on those with an increase of more than 25 per cent, decomposing the increase into changes due to the extensive margin, intensive margin and a covariance term.

Panel a of Figure 3 shows the distribution of these percentage changes across categories. Thirty categories, all of which belong to the broader product groupings of staples or household supplies, experienced an increase in quantity bought of more than 25 per cent (see panel b for the list of these). The quantity of soap purchased more than doubled over this period relative to the previous year. Other categories that experienced significant spikes in purchases include ambient soup (75 per cent higher), cold treatments (64 per cent), rice and noodles (54 per cent) and dried pasta (49 per cent). Given the ‘just‐in‐time’ nature of UK grocery supply chains,^15^ it is not surprising that there were widespread stories of shortages in these categories. The 30 categories that experienced an increase in quantity of more than 25 per cent jointly account for 70 per cent of the overall increase in spending on household supplies and 53 per cent of the overall increase in spending on staples over this period.

#### 4.1.1 Extensive versus intensive margin changes

Was the spike in category demands driven by households purchasing more often or by them buying larger quantities conditional on purchasing? To answer this, we decompose the change in quantity purchased in the four‐week period running up to 23 March in 2020 relative to the same period in 2019. Let $Q_{jy}$ denote average daily quantity purchased of category j in year y={2019,2020} over this four‐week period; this is given by

$$\begin{array}{cc} Q_{jy} & =\frac{1}{N}\sum_{i}\sum_{t\in P_{y}}q_{ijt}, \end{array}$$

where $q_{ijt}$ denotes the total quantity of j purchased by household i on date t, $P_{y}$ indexes the set of 28 dates over the four‐week period in year y, and N denotes the number of households in the sample multiplied by the number of days (28) in the period. Let $N_{jy}^{+}=\sum_{i}\sum_{t\in P_{y}}1\left\{q_{ijt}>0\right\}$ denote the number of household‐days on which category j was bought. We can rewrite $Q_{jy}=E_{jy}\times Q_{jy}^{c}$, where $E_{jy}=N_{jy}^{+}/N$ is the fraction of household‐days on which category j was bought and $Q_{\mathrm{jy}}^{c}=\left(1/N_{\mathrm{jy}}^{+}\right)\sum_{i}\sum_{t\in P_{y}}q_{\mathrm{ijt}}$ is the average quantity bought, conditional on the category being purchased. Defining $\Delta X=X_{2020}-X_{2019}$, we can then write

$$\Delta Q_{j}=\underset{\text{extensive}\;\text{margin}}{\underset{︸}{Q_{j2019}^{c}\times \Delta E_{j}}}+\underset{\text{intensive}\;\text{margin}}{\underset{︸}{E_{j2019}\times \Delta Q_{j}^{c}}}+\underset{\text{covariance}}{\underset{︸}{\Delta E_{j}\times \Delta Q_{j}^{c}}}.$$

Panel b of Figure 3 shows the contribution that each of these components makes to the demand spike in the 30 categories that experience overall increases in demand of more than 25 per cent. We report these numbers in Table A.4 of the online appendix. Two things are evident from the figure.

First, the extensive margin contributes more towards the demand spike than the intensive margin: in 27 of the 30 categories, the extensive margin accounts for at least 50 per cent of the spike. On average across the categories, an increase in the fraction of household‐days on which the category was bought accounts for 70 per cent of the spike in demand. Second, the increase in quantity attributable to the intensive margin is roughly similar across categories – for most categories, the increase in quantity, conditional on buying, ranges between 5 and 15 per cent. Therefore, the extensive margin change was relatively more important in driving demand spikes of those categories that experienced the largest overall increases.

#### 4.1.2 Did households make multiple purchases or shop more often?

An increase in the fraction of household‐days on which the category was purchased was the primary driver of the large quantity spikes in key storable categories. To what extent was this driven by some households making more multiple purchases of this category (on different days), or by more households than usual choosing to buy the category over the March period? Relatedly, was the increase driven by households visiting the store more often in general, or being more likely to buy storable foods and supplies conditional on visiting the store? To answer these questions, we conduct two further decompositions.

First, we decompose the increase in the fraction of household‐days on which the category was purchased into the change due to an increase in the fraction of households buying the category at least once over the period, and the part due to a change in the *number* of times these households bought the category over the four‐week period. Let $N_{jy}^{+,hh}=\sum_{i}1\left\{\max_{t\in P_{y}}q_{ijt}>0\right\}$ denote the number of households that buy category j at least once over period y, $E_{\mathrm{jy}}^{\mathrm{hh}}=N_{\mathrm{jy}}^{+,\mathrm{hh}}/\left(N/28\right)$ denote the corresponding fraction of households, and $E_{\mathrm{jy}}^{\mathrm{mult}}=N_{\mathrm{jy}}^{+}/\left(N_{\mathrm{jy}}^{+,\mathrm{hh}}\times 28\right)$ denote, for those households that buy at least once, the average fraction of days on which they buy. We can then write $E_{jy}=E_{jy}^{mult}\times E_{jy}^{hh}$ and decompose as follows:

$$\Delta E_{j}=\underset{\text{new}\;\text{shoppers}}{\underset{︸}{E_{j2019}^{mult}\times \Delta E_{j}^{hh}}}+\underset{\text{multiple}\;\text{purchases}}{\underset{︸}{E_{j2019}^{hh}\times \Delta E_{j}^{mult}}}+\underset{\text{covariance}}{\underset{︸}{\Delta E_{j}^{hh}\times \Delta E_{j}^{mult}}}.$$

Table A.4 in the online appendix summarises the results for the top 30 categories. It shows that the extensive margin change was primarily driven by an increase in the number of unique households buying the category, which accounted for 76 per cent of the extensive margin response, on average. Thus the biggest driver of the demand spikes was the fact that more households than usual chose to buy these categories over this period, rather than some households repeatedly buying them.

Second, we decompose the increase in the fraction of household‐days on which the category was purchased into the fraction due to households making more trips to the store to buy anything (shopping frequency) versus households being more likely to buy the category conditional on visiting the store (purchase incidence). Let $N_{y}^{+,trips}=\sum_{i}\sum_{t\in P_{y}}1\{\max_{j}q_{ijt}$ >0} denote the number of household‐days on which *any* category was purchased over the period y – i.e. the number of shopping trips undertaken by households in the sample – $E_{jy}^{incid}=N_{jy}^{+}/N_{y}^{+,trips}$ denote the fraction of trips on which the category was purchased, and $E_{jy}^{trips}=N_{y}^{+,trips}/N$ denote the fraction of household‐days on which a shopping trip was undertaken. We can then write $E_{jy}=E_{jy}^{incid}\times E_{jy}^{trips}$ and decompose this into

$$\Delta E_{j}=\underset{\text{shopping}\;\text{frequency}}{\underset{︸}{E_{j2019}^{incid}\times \Delta E_{j}^{trips}}}+\underset{\text{purchase}\;\text{incidence}}{\underset{︸}{E_{j2019}^{trips}\times \Delta E_{j}^{incid}}}+\underset{\text{covariance}}{\underset{︸}{\Delta E_{j}^{trips}\times \Delta E_{j}^{incid}}}.$$

Table A.4 presents the results of this decomposition. We find that the extensive margin change is almost entirely driven by increases in the purchase incidence for these categories, rather than by households visiting a store more frequently. Increased probability of buying, *conditional on visiting a store*, accounts for, on average, 90 per cent of the extensive margin change for the categories that saw the largest spikes in demand. The period of hoarding of key storable categories was primarily driven by more households than normal choosing to buy these categories once, and not by increased shopping frequency, nor households buying larger quantities conditional on purchasing.

This is relevant when we consider how changes in shopping patterns may affect the propensity for future bouts of hoarding. In the online appendix, we show how the average number of shopping trips made by households varies over 2019–20 (see Figure A.3). We find that there was a small increase in the number of shopping trips during the four‐week period leading up to lockdown (23 March 2020), followed by a 10–15 per cent decline in shopping frequency once lockdown restrictions were introduced. The fact that the quantity spikes were not driven by increased shopping frequency suggests that further spates of hoarding could still occur, even during periods when households are shopping less frequently than normal.

#### 4.1.3 Cross‐category correlation

To what extent were household‐level spikes in demand correlated across categories? To answer this, we compute household‐level changes in average daily quantity purchased in the four weeks running up to 23 March in 2020 relative to the same period in 2019. For each of the 30 categories that exhibited the biggest demand spikes, we take the pairwise correlation in these changes across households. 434 of the 435 pairwise correlations are positive. The median correlation coefficient is 0.1. The categories with the largest correlation coefficients (above 0.25) are: ‘dry pasta’ and ‘ambient cooking sauces’; ‘canned pasta products’ and ‘baked beans’; and ‘tomato products’ and ‘dry pasta’. Overall, there is a consistent but modest correlation in household‐level demand changes across categories. If a household raised its demand for one category, it is likely that it also raised its demand across others, but the predictive power of changes in demand for one category for changes in a specific second category is low.^16^

### 4.2 Heterogeneity by socio‐economic group

A common concern raised by the media and policymakers in the run‐up to lockdown was that some vulnerable households may be failing to access the products they need. In this section, we explore differences in purchase dynamics across socio‐economic groups. We use the social grade of the household, which is based on the occupation of the head of the household and is a good proxy for the household's permanent income. Table A.2 in the online appendix lists the five socio‐economic groups and the share of households in each. ‘AB’ is the highest group, consisting of households with a head who is occupied in a managerial, administrative or professional role; ‘E’ is the lowest group, consisting of those households with non‐working heads – for example, state pensioners, casual and lowest‐grade workers, and unemployed people with only state benefit income.

Figure A.4 in the online appendix shows how spending on staples, household supplies, discretionary calories and perishables evolved over January to August 2020 for each socio‐economic group. All five groups exhibit a substantial increase in their spending on staples and household supplies during the four‐week period prior to lockdown. However, there is a clear gradient in the size of these spikes: for staples, over the four weeks up to 23 March, daily spending was 31 per cent above its prior 2020 average for AB households and 18 per cent higher for E households. The increase in spending on household supplies was 27 per cent for AB households and 18 per cent for E households.

**FIGURE 4:**
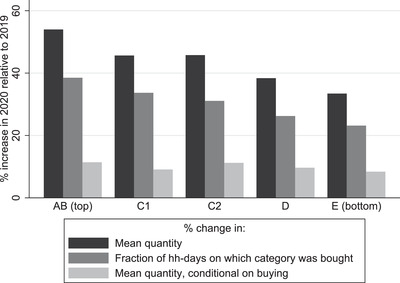
Average change across product categories with largest demand spikes, by socio‐economic group *Note*: The figure shows the unweighted mean (across the top 30 categories shown in panel b of Figure 3) percentage change in quantity, fraction of household‐days on which the category was bought, and quantity conditional on buying, for each socio‐economic group.

These patterns are also evident at the product category level. In Figure 4, we show the average (across the top 30 categories) increase in quantity purchased for the different socio‐economic groups. The figure also shows the average (again, across the top 30 categories) contribution made by changes in (i) the number of household‐days on which the category was bought and (ii) the quantity conditional on buying. On average, AB households increased the quantity bought of these categories by 54 per cent, compared with 33 per cent for households in the lowest group. This gradient is almost entirely driven by changes along the extensive margin; changes in conditional quantity are similar across the socio‐economic groups. The greater propensity of higher socio‐economic households to hoard was therefore driven by the fact they increased the probability of buying these categories by more than other households.

A socio‐economic gradient in spending changes persists through the first few months of the pandemic: households from higher socio‐economic groups, on average, have higher levels of spending (albeit lower than the March peak) than lower socio‐economic households through to the start of August. One likely driver of this is differences in the importance of food consumed in dine‐in restaurants, prior to the pandemic, across these groups. For instance, according to the LCFS, in 2018, households with a head in a highly skilled occupation consumed 10.6 per cent of their calories in restaurants, while this share was only 6.8 per cent for those with a head in an unskilled occupation. Households in higher socio‐economic groups would therefore have to increase their calories from groceries by more than lower socio‐economic households to compensate for the shutdown in dine‐in hospitality over the pandemic. Another possible reason for this pattern would arise if households from lower socio‐economic groups are more likely to be in occupations that cannot be carried out from home (as documented by Carvalho et al. (2020) for Spain), meaning they consume more food not brought into the home (and therefore not recorded in our data) and less household supplies than groups who work from home. Systematically explaining differences in spending patterns over the pandemic, and assessing the overall impact on calories and diet quality, are important avenues for future research.

## 5 LESSONS FOR POLICY

During the run‐up to the first national lockdown, there were reports of shortages in many stores. These led to calls for policy intervention to tackle the shortages.

In the final few days before the lockdown, supermarkets introduced limits on the number of units of a product households could buy per transaction.^17^ By the time quantity limits came into effect, demand was already returning to normal levels. A resurgence of case numbers and the threat of a second nationwide lockdown led some supermarkets to reintroduce quantity limits in September 2020. We have shown that much of the demand spike in March 2020 was driven by more households than usual choosing to buy storable food and supplies on at least one of their shopping trips over this period, rather than buying much larger quantities.^18^ This suggests that quantity limits may not prevent the shortages induced by the demand spikes.

**FIGURE 5:**
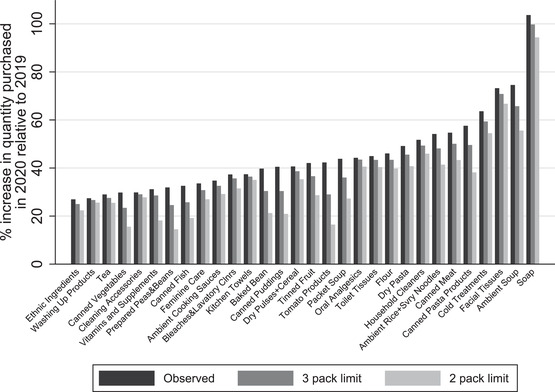
Change in quantity spikes under counterfactual quantity limits *Note*: The black bars show the percentage increase in quantity purchased for each category between the four‐week period ending 22 March in 2019 and the same period in 2020. The dark and light grey bars show the analogous, counterfactual increases if households were limited to buying no more than three and no more than two packs per transaction, respectively.

In Figure 5, we graph the quantity spikes for the top 30 categories, as well as what they would have been if a limit of three or two units per transaction were in place over the whole four weeks running up to lockdown. This assumes that households do not circumvent the limits by purchasing different UPCs in the same category or by undertaking more regular grocery store visits. It should therefore be viewed as an upper bound for the impact of the quantity limits. The graph shows that the limits are likely to have had only a moderate effect in reducing demand; even with the limits, there are still very large increases in demand for the majority of categories – for instance, were the two‐pack limit in place, the average increase in quantity in the 30 categories would have been 34 per cent rather than 44 per cent; among the eight categories that experienced spikes of more than 50 per cent, the average quantity increase would have been 55 per cent rather than 66 per cent.

This begs the question ‘So what can be done about shortages arising from hoarding?’. Another policy implemented by several supermarkets in March 2020 was to have dedicated shopping hours for the elderly and other vulnerable consumers. If these were timed to coincide with deliveries of new stock, this may help to ensure that vulnerable consumers were able to procure the supplies that they needed. We are not able to clearly identify ‘vulnerable’ consumers in our data. However, we do find that those in the lowest socio‐economic group – who are more likely to be older and less financially secure – did not increase their purchase frequency by nearly as much as richer households, and did not offset this through buying larger quantities conditional on purchasing. This means that they built up smaller precautionary stocks than more well‐off households.

## 6 SUMMARY AND CONCLUSIONS

In this paper, we provide new evidence on consumer purchase dynamics and hoarding during the early phase of the COVID‐19 pandemic. We show that a number of storable categories experienced dramatic spikes in demand, and that this was primarily driven by more consumers than usual buying products in these categories on at least one of their shopping trips over this period, rather than increasing the amount they bought on any particular trip. Unusually high demands were widespread across households, although higher socio‐economic households increased the quantities they bought by more than lower socio‐economic households.

The ongoing pandemic, the risk of extreme weather and natural disasters due to climate change, and the likelihood of trade disruptions due to heightened international political uncertainty mean further periods of acute uncertainty and sharp demand rises are increasingly likely. Shortages due to the COVID‐19 first wave led to serious concerns over the sustainability of the food system and people's ability to access the products they need. Retailers and policymakers will need to prepare for further bouts of panic buying by improving the resilience of supply chains and designing policy that ensures access for the most vulnerable. Our findings provide invaluable evidence on the nature of the demand spike – it was concentrated in a relatively small set of products, but was widespread across households – which will hopefully improve preparations for future hoarding episodes.

## Supporting information

• Appendix

## Footnotes

1 For instance, see https://eu.usatoday.com/story/money/2020/03/29/covid‐19‐stockpiling‐food‐buy‐if‐youre‐budget/5080290002/ in the US and https://www.ft.com/content/737a9a24‐68ea‐11ea‐a3c9‐1fe6fedcca75 in the UK.

2 On 21 March 2020, the UK Secretary of State for Environment, Food and Rural Affairs urged consumers to ‘Be responsible when you shop and think of others’ – see https://www.reuters.com/article/health‐coronavirus‐britain‐shopping‐idINL9N29I02G.

3 See https://www.gov.uk/government/news/covid‐19‐cma‐approach‐to‐essential‐business‐cooperation.

4 For example, see Baker et al. (2020a) and Cox et al. (2020) for the US, and Hacioglu, Känzig and Surico (2020) for the UK.

5 We show that the number of shopping trips per four‐week period dropped by 15 per cent, relative to the pre‐pandemic period, following the beginning of lockdown.

6 See, for instance, Alexander and Karger (2020), Andersen et al. (2020), Baker et al. (2020b), Bounie, Camara and Galbraith (2020), Carvalho et al. (2020), Chen, Qian and Wen (2020), Chetty et al. (2020), Chronopoulos, Lukas and Wilson (2020) and Coibion, Gorodnichenko and Weber (2020).

7 In years preceding the pandemic, the scanner data account for approximately 40 per cent of household expenditures on goods and 15 per cent of total household expenditures on both goods and services (see Jaravel (2019)).

8 See Leicester and Oldfield (2009a and 2009b).

9 This is true in the 2018 data, where the median weekly food expenditure recorded by households in the Kantar data is four‐fifths that in the LCFS.

10 Department of Health and Social Care, 2020.

11 See Table A.3 in the online appendix for a full list of product categories.

12 Jaravel and O'Connell (2020b) show that month‐to‐month inflation for fast‐moving consumer goods in the first month of lockdown was 2.4 per cent. Prices then gradually declined towards pre‐COVID levels (see Jaravel and O'Connell (2020a)). The increase in spending on fast‐moving consumer goods from the beginning of lockdown to the end of our data (a period when households were urged to work from home) compared with 2019 was 11 per cent.

13 See, for instance, *The Sun*'s report on 15 March speculating over the introduction of food rationing if greedy shoppers ignore warnings to stop panic buying (https://www.thesun.co.uk/news/11179394/food‐rationing‐to‐stop‐coronavirus‐panic‐buying/).

14 Since 2020 was a leap year, the four weeks cover 23 February to 22 March in 2019 and 24 February to 22 March in 2020.

15 Garnett, Doherty and Heron, 2020.

16 Note that if instead we compare 24 February to 22 March 2020 with the four‐week period 27 January to 23 February 2020, the pattern is very similar.

17 For instance, on 19 March 2020, Tesco, the largest supermarket chain, introduced a limit that people could purchase no more than three of any item per visit. This followed the adoption of similar restrictions by Sainsbury's (another large chain) the day before.

18 There are exceptions. In September 2020, the *Daily Mail* reported on someone who had just purchased 400 tins and 700 nappies due to fear of rationing (https://www.dailymail.co.uk/femail/article‐8789051/Mother‐two‐slammed‐stockpiling‐shopping‐JANUARY.html).
