# Supplementary material for: Preparing for a pandemic: spending dynamics and panic buying during the COVID‐19 first wave
Source: Fisc Stud. 2021 Jun 8;42(2):249–64. doi: 10.1111/1475-5890.12271 (PMC8209877; doi:10.1111/1475-5890.12271)
Supplement: Supplementary file 1 — • Appendix [file FISC-42-249-s001.pdf]

Appendix

Preparing for a pandemic: spending dynamics and panic buying during the COVID-19 first wave

Martin O'Connell, Áureo de Paula and Kate Smith

A Additional tables and figures

Table A.1: *Household demographics*

	Kantar	LCFS
Region		
England - North (%)	28.1	25.9
	[27.4, 28.8]	[24.6, 27.2]
England - Midlands (%)	17.9	16.6
	[17.3, 18.5]	[15.5, 17.7]
England - South and East (%)	44.8	46.5
	[44.0, 45.6]	[45.0, 48.0]
Scotland (%)	9.2	11.0
	[8.8, 9.7]	[10.2, 11.8]
Employment status of household head		
Full-time (%)	38.6	43.3
	[37.8, 39.3]	[41.8, 44.8]
Part-time (%)	20.2	9.9
	[19.5, 20.8]	[9.0, 10.7]
Self-employed (%)	-	8.4
		[7.6, 9.3]
Unemployed (%)	1.6	1.8
	[1.4, 1.8]	[1.4, 2.3]
Retired or not working (%)	39.7	36.5
	[38.9, 40.4]	[35.1, 38.0]
Socio-economic group		
Highly skilled (%)	20.7	22.2
	[20.0, 21.5]	[20.6, 23.8]
Semi-skilled (%)	59.9	57.8
	[59.0, 60.8]	[55.9, 59.7]
Unskilled (%)	19.4	20.1
	[18.7, 20.1]	[18.5, 21.6]

Note: The table shows the shares of households in the Kantar Worldpanel and the Living Costs and Food Survey (LCFS) in various demographic groups. Numbers are shown for the most recently available data for the LCFS, which are 2018–19, and for the households in our 2019–20 sample from the Kantar data. The self-employed are not distinguished from employees in the Kantar data. Socio-economic status is based on the occupation of the head of the household and is shown for the set of non-retired households – this is not recorded for non-retired households in the LCFS. Highly skilled corresponds to AB, semi-skilled to C1 and C2, and unskilled to DE listed in Table A.2. We use the weights provided with the LCFS data to calculate the shares for this data set. 95 per cent confidence intervals are shown below each share.

Figure A.1: *Spending on food and non-alcoholic drinks, 2011–18*

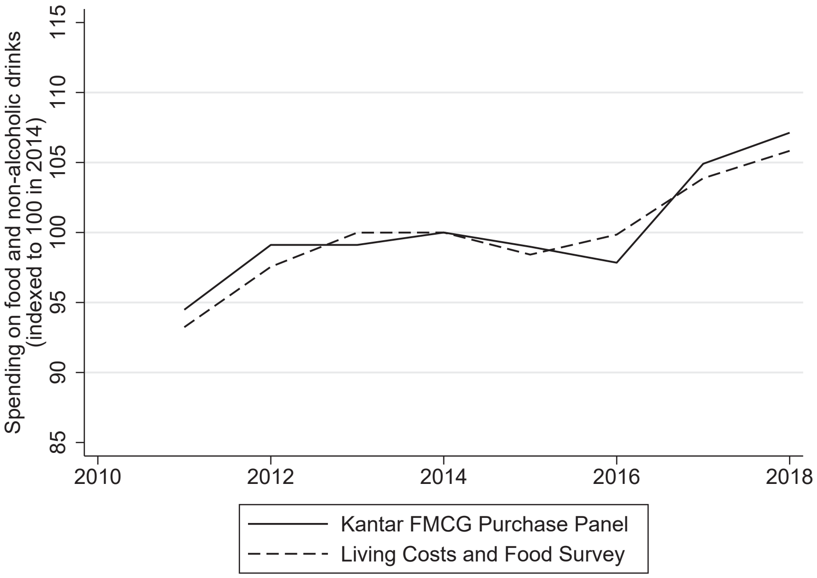

Note: The solid line shows the mean spending on food and non-alcoholic drinks made by households in the Kantar FMCG Purchase Panel over the period 2011–18, indexed to 100 in 2014. The dashed line shows the mean spending on food and non-alcoholic drinks made by households in the Living Costs and Food Survey over the period 2011–18, indexed to 100 in 2014.

Figure A.2: *Aggregate grocery spending*

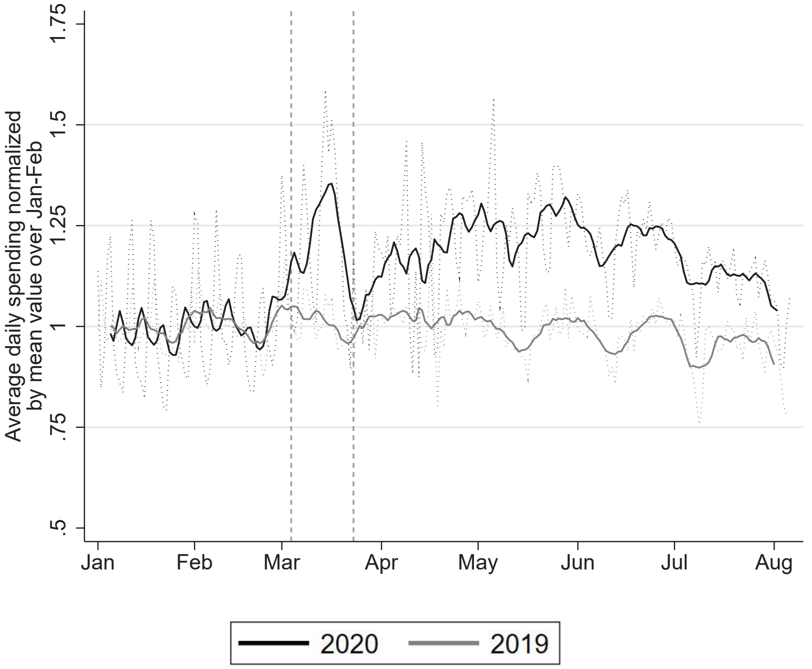

Note: The dotted lines show daily expenditure after removing day of the week and holiday effects. The solid black line shows a nine-day moving average through these points. In each case, the line is normalised by the mean value over January and February. The vertical dashed lines indicate the announcement of the UK's coronavirus action plan on 3 March and the beginning of lockdown on 23 March.

Table A.2: *Socio-economic groups*

Socio-economic group	Description	% of households
AB (top)	Managerial, administrative or professional	21.0
C1	Supervisory or clerical and junior managerial, administrative or professional	40.0
C2	Skilled manual workers	17.6
D	Semi-skilled and unskilled manual workers	13.3
E (bottom)	State pensioners, casual and lowest-grade workers, unemployed with state benefits only	8.0

Note: The table shows the description of the socio-economic groups that we use and the percentage of households belonging to each group.

Table A.3: *Product categories*

	Product category	Share of spending in 2019 (%)	% increase during hoarding
**Staples**		16.16	
	Ambient Condiments	0.20	15.11
	Ambient Cooking Sauces	0.68	34.75
	Ambient Rice + Savoury Noodles	0.57	54.16
	Ambient Soup	0.30	74.54
	Ambient Salad Accompaniment	0.24	13.32
	Baked Beans	0.36	39.75
	Breakfast Cereals	1.59	19.41
	Canned Fish	0.54	32.59
	Canned Meat	0.26	54.70
	Canned Pasta Products	0.09	57.57
	Canned Puddings	0.03	40.51
	Canned Vegetables	0.19	29.76
	Cooking Oils	0.32	16.89
	Dry Pasta	0.21	49.14
	Dry Pulses + Cereal	0.16	40.62
	Ethnic Ingredients	0.21	26.95
	Flour	0.10	46.04
	Food Drinks	0.17	7.16
	Frozen Fish	0.95	18.21
	Frozen Meat	1.25	17.97
	Frozen Pizzas	0.57	14.26
	Frozen Potato Products	0.83	16.32
	Frozen Ready Meals	0.71	7.58
	Frozen Savoury Bakery	0.25	17.99
	Frozen Vegetables	0.78	20.26
	Herbal Tea	0.11	12.28
	Herbs + Spices	0.20	18.41
	Instant Coffee	0.85	7.99
	Instant Hot Snacks	0.23	21.74
	Liquid + Ground Coffee + Beans	0.47	22.75
	Meat Extract	0.38	24.99
	Other Frozen Foods	0.16	8.06
	Packet Soup	0.10	43.82
	Pickles, Chutneys + Relish	0.09	2.82
	Prepared Peas + Beans	0.15	31.90
	Preserves and spreads	0.48	12.01
	Sour + Speciality Pickles	0.12	–1.36
	Sweet + Savoury Mixes	0.10	22.06
	Table Sauces	0.30	13.55
	Tea	0.47	28.98
	Tinned Fruit	0.16	42.06
	Tomato Products	0.25	42.29

Table A.3 cont.

	Product category	Share of spending in 2019 (%)	% increase during hoarding
**Household supplies**		14.04	
	Air Fresheners	0.31	–13.67
	Anti-Diarrhoeals	0.15	14.15
	Bath + Shower Products	0.38	10.46
	Batteries	0.24	13.41
	Bin Liners	0.20	17.58
	Bleaches + Lavatory Cleaners	0.26	37.29
	Body Sprays	0.05	–5.47
	Cleaning Accessories	0.14	29.84
	Cold Treatments	0.27	63.59
	Cotton Wool	0.04	11.60
	Dental Products	0.81	11.10
	Deodorants	0.41	10.30
	Electric Light Bulbs	0.04	–33.88
	Eye Care	0.04	7.13
	Fabric Conditioners	0.40	16.42
	Facial Tissues	0.25	73.21
	Feminine Care	0.34	33.59
	First Aid Dressings	0.03	13.00
	Foot Preparations	0.07	–17.29
	Hair Colourants	0.14	4.73
	Hair Conditioners	0.18	–2.65
	Hair Styling	0.07	–19.60
	Hairsprays	0.07	–0.37
	Household Cleaners	0.57	51.71
	Household Food Wraps	0.22	5.46
	Kitchen Towels	0.39	37.39
	Machine-Wash Products	0.92	15.03
	Moist Wipes	0.13	20.73
	Oral Analgesics	0.25	44.24
	Other Healthcare	0.25	15.14
	Other Household	0.17	11.03
	Pet Food	2.67	16.69
	Pot Pourri + Scented Candles + Oil	0.08	–25.62
	Shampoo	0.30	2.26
	Shaving	0.22	–14.17
	Skincare	0.63	11.78
	Soap	0.19	103.66
	Sun Care	0.09	–26.55
	Toilet Tissues	1.28	44.94
	Vitamins and Supplements	0.36	31.18
	Washing-Up Products	0.45	27.39

Table A.3 cont.

	Product category	Share of spending in 2019 (%)	% increase during hoarding
**Discretionary calories**		27.38	
	Ambient Cakes + Pastries	1.54	–25.50
	Beer + Lager	1.81	7.40
	Chilled Cakes	0.96	–6.05
	Chocolate Confectionery	2.61	–3.31
	Cider	0.50	5.62
	Crisps	0.90	10.55
	Fruit Juice	0.87	8.47
	Home Baking	0.82	20.82
	Ice Cream	1.40	–9.95
	Long-Life Milk and Desserts	0.40	17.64
	Milk Drinks	0.17	–1.70
	Mineral Water	0.45	–2.57
	Mixers	0.18	10.30
	Nuts	0.62	2.44
	Popcorn	0.10	10.80
	Savoury Biscuits	0.72	8.59
	Savoury Snacks	1.04	3.98
	Soda	2.28	7.36
	Spirits	2.64	13.00
	Sugar Confectionery	0.83	–1.86
	Sweet Biscuits	2.02	7.40
	Total Fruit Squash	0.54	14.12
	Wine	3.99	7.64
**Perishables**		42.41	
	Butter and Fats	1.46	20.06
	Chilled Burgers + Grills	0.29	13.08
	Chilled Cooking Sauces	0.08	6.73
	Chilled Deli	0.34	–2.87
	Chilled Pizza + Bases	0.51	–1.93
	Chilled Prepared Fruit + Vegetables	0.92	–3.56
	Chilled Prepared Salad	0.48	–2.97
	Chilled Ready Meals	2.52	0.02
	Cooked Meats	2.12	–12.17
	Cooked Poultry	0.51	1.51
	Eggs	0.77	7.40
	Fresh Bacon	1.15	2.16
	Fresh Beef	2.01	24.63
	Fresh Cream	0.35	8.66
	Fresh Flavoured Meats	0.16	15.44
	Fresh Lamb	0.46	2.29
	Fresh Pasta	0.15	24.02
	Fresh Pork	0.63	0.58
	Fresh Poultry	2.19	11.25

Table A.3 cont.

Product category	Share of spending in 2019 (%)	% increase during hoarding
Fresh Sausages	0.92	12.47
Fresh Soup	0.09	5.48
Fruit	4.93	–5.44
Morning Goods	1.70	–0.97
Other Chilled Convenience	0.39	–12.01
Pre-Packed Fresh Meat + Vegetables + Pastry	1.71	3.36
Shellfish	0.18	0.23
Total Bread	1.67	2.35
Total Cheese	2.85	11.13
Total Milk	2.79	3.38
Vegetables	5.21	4.82
Wet/Smoked Fish	0.92	–1.83
Yoghurt	1.96	–4.58

Note: The table lists the 138 products that together comprise all fast-moving consumer goods, grouped into staples, household supplies, discretionary calories and perishables. The second column shows the share of total spending on fast-moving consumer goods in 2019 accounted for by each category. The third column shows the percentage change in average quantity purchased between the four-week period ending 22 March 2020 and the same period in 2019.

Table A.4: *Purchase dynamics, top 30 categories*

(1)	(2)	(3)	(4)	(5)	(6)	(7)	(8)	(9)
		% of overall change due to:			% of extensive margin due to:		% of extensive margin due to:	
Category	$\frac{\Delta Q_{jy}}{Q_{j2019}}$	Ext.	Int.	Cov.	No.hhs	Mult.	Trips	Incid.
Soap	103.7	85.0	8.0	7.0	68.7	19.5	2.6	95.2
Ambient Soup	74.5	73.6	17.0	9.4	84.9	10.3	4.2	93.6
Facial Tissues	73.2	75.8	15.6	8.6	78.6	14.9	4.2	93.7
Cold Treatments	63.6	85.0	9.7	5.3	87.3	8.6	4.3	93.6
Canned Pasta Products	57.6	69.9	21.5	8.6	101.9	-1.4	5.7	92.1
Canned Meat	54.7	62.9	27.6	9.5	82.2	13.9	6.7	91.2
Ambient Rice + Svry Noodles	54.2	61.7	28.7	9.6	63.9	29.7	6.9	91.0
Household Cleaners	51.7	72.7	19.8	7.5	57.7	34.7	6.1	91.7
Dry Pasta	49.1	58.6	32.2	9.3	69.5	25.4	8.0	89.9
Flour	46.0	68.5	23.9	7.6	64.9	29.1	7.3	90.6
Toilet Tissues	44.9	59.5	32.0	8.5	58.3	36.1	8.6	89.3
Oral Analgesics	44.2	77.2	17.0	5.8	88.1	9.1	6.8	91.1
Packet Soup	43.8	103.2	-2.2	-1.0	87.8	8.7	5.1	92.7
Tomato Products	42.3	59.7	32.2	8.1	63.8	31.2	9.2	88.8
Tinned Fruit	42.1	66.5	26.2	7.3	94.8	4.1	8.3	89.7
Dry Pulses + Cereal	40.6	38.5	53.1	8.3	78.3	19.3	14.8	83.3
Canned Puddings	40.5	76.2	18.2	5.6	110.4	-7.7	7.5	90.4
Baked Bean	39.8	69.1	24.2	6.7	71.5	23.8	8.4	89.5
Kitchen Towels	37.4	85.4	11.1	3.5	77.7	17.9	7.2	90.7
Bleaches + Lavatory Clnrs	37.3	97.8	1.6	0.6	65.0	28.3	6.3	91.5
Ambient Cooking Sauces	34.7	49.5	43.1	7.4	52.9	43.1	13.4	84.6
Feminine Care	33.6	67.1	26.8	6.1	54.8	40.2	10.3	87.7
Canned Fish	32.6	64.5	29.4	6.2	77.8	19.1	11.0	87.0
Prepared Peas + Beans	31.9	62.7	31.1	6.2	88.9	9.4	11.6	86.4
Vitamins and Supplements	31.2	87.4	9.9	2.7	84.5	12.6	8.5	89.4
Cleaning Accessories	29.8	72.8	22.4	4.9	79.3	17.7	10.6	87.3
Canned Vegetables	29.8	54.4	39.2	6.4	86.4	11.9	14.3	83.8
Tea	29.0	72.3	22.9	4.8	64.6	31.2	11.0	87.0
Washing Up Products	27.4	86.6	10.8	2.6	69.7	26.0	9.7	88.2
Ethnic Ingredients	26.9	42.6	51.5	5.9	81.6	16.8	20.1	78.1

Note: Column 2 shows the percentage change in average quantity purchased between the four-week period ending 22 March 2020 and the same period in 2019. Columns 3–5 decompose this change into the shares attributable to the extensive margin (3), the intensive margin (4) and a covariance term (5). Columns 6 and 7 further decompose the change in the extensive margin into the shares attributable to the change in the number of unique households that bought the category (6) and the number of times each household bought the category conditional on buying at least once (7). Columns 8 and 9 conduct a separate decomposition, also on the extensive margin, but into the shares attributable to households making more shopping trips (8) and to the probability of buying the category conditional on visiting the store (9).

Figure A.3: *Average number of shopping trips per four-week period*

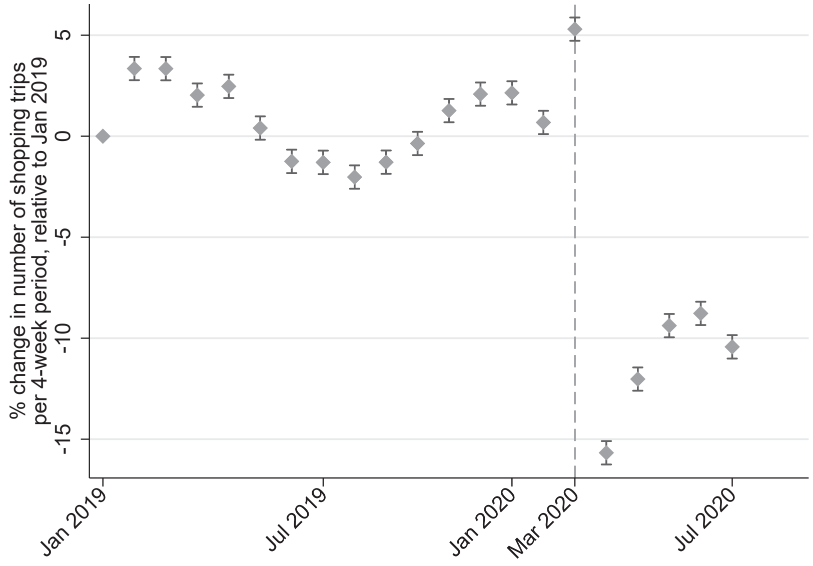

Note: For each household-four-week-period, we calculate the number of days that the household records buying groceries. The markers show the average difference (relative to January 2019) in the log of this variable in each four-week period from January 2019 to July 2020. The vertical dashed line shows the four-week period prior to the beginning of lockdown.

Figure A.4: *Aggregate spending, by socio-economic group*

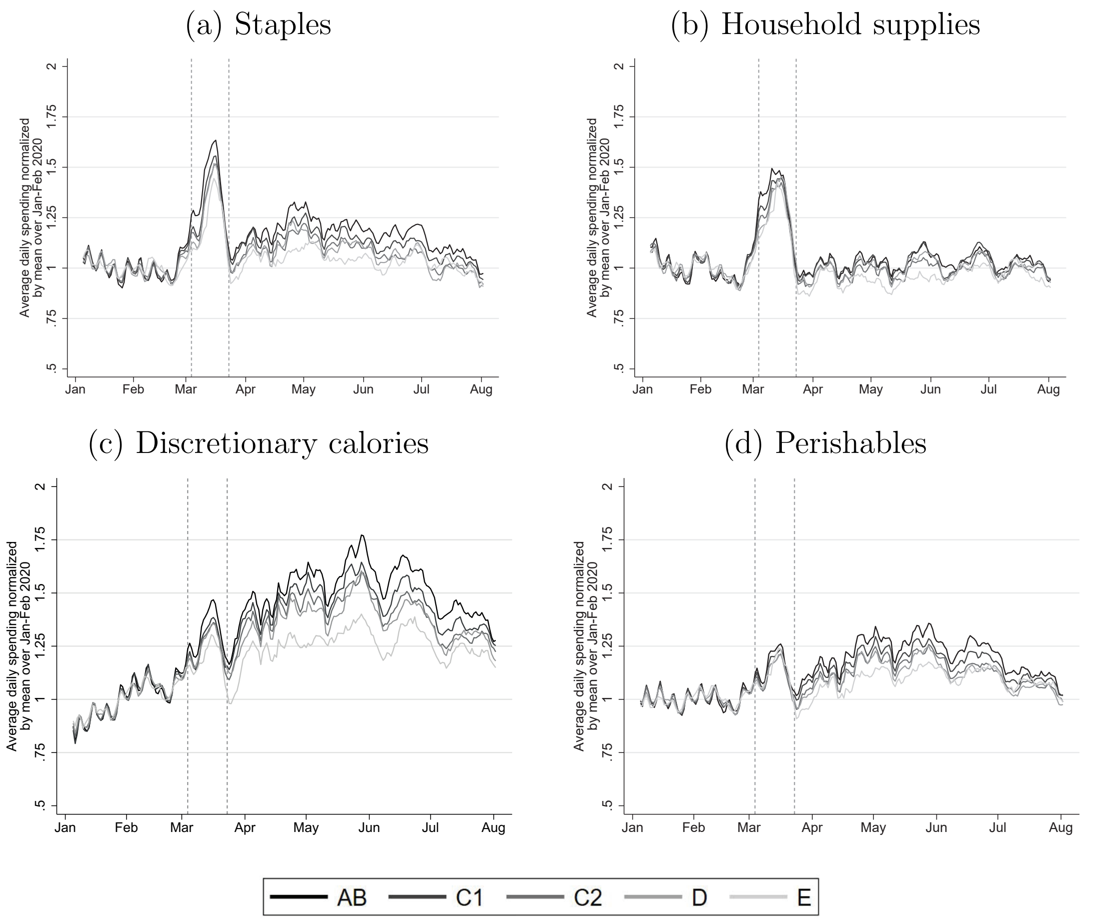

Note: The panels show total daily expenditure on staples, household supplies, discretionary calories and perishables, by socio-economic group. See Table A.3 for a list of the product categories in each grouping. The lines show a nine-day moving average through daily expenditure, after removing day of the week and holiday effects. In each case, the line is normalised by the mean value over January and February 2020. The vertical dashed lines indicate the announcement of the UK's coronavirus action plan on 3 March 2020 and the beginning of lockdown on 23 March.
